# Integrating GPT-Based AI into Virtual Patients to Facilitate Communication Training Among Medical First Responders: Usability Study of Mixed Reality Simulation

**DOI:** 10.2196/58623

**Published:** 2024-12-11

**Authors:** Rodrigo Gutiérrez Maquilón, Jakob Uhl, Helmut Schrom-Feiertag, Manfred Tscheligi

**Affiliations:** 1 Center for Technology Experience AIT - Austrian Institute of Technology Vienna Austria; 2 Department of Artificial Intelligence and Human Interfaces Paris Lodron University of Salzburg Salzburg Austria

**Keywords:** medical first responders, verbal communication skills, training, virtual patient, generative artificial intelligence, GPT, large language models, prompt engineering, mixed reality

## Abstract

**Background:**

Training in social-verbal interactions is crucial for medical first responders (MFRs) to assess a patient’s condition and perform urgent treatment during emergency medical service administration. Integrating conversational agents (CAs) in virtual patients (VPs), that is, digital simulations, is a cost-effective alternative to resource-intensive human role-playing. There is moderate evidence that CAs improve communication skills more effectively when used with instructional interventions. However, more recent GPT-based artificial intelligence (AI) produces richer, more diverse, and more natural responses than previous CAs and has control of prosodic voice qualities like pitch and duration. These functionalities have the potential to better match the interaction expectations of MFRs regarding habitability.

**Objective:**

We aimed to study how the integration of GPT-based AI in a mixed reality (MR)–VP could support communication training of MFRs.

**Methods:**

We developed an MR simulation of a traffic accident with a VP. ChatGPT (OpenAI) was integrated into the VP and prompted with verified characteristics of accident victims. MFRs (N=24) were instructed on how to interact with the MR scenario. After assessing and treating the VP, the MFRs were administered the Mean Opinion Scale-Expanded, version 2, and the Subjective Assessment of Speech System Interfaces questionnaires to study their perception of the voice quality and the usability of the voice interactions, respectively. Open-ended questions were asked after completing the questionnaires. The observed and logged interactions with the VP, descriptive statistics of the questionnaires, and the output of the open-ended questions are reported.

**Results:**

The usability assessment of the VP resulted in moderate positive ratings, especially in habitability (median 4.25, IQR 4-4.81) and likeability (median 4.50, IQR 3.97-5.91). Interactions were negatively affected by the approximately 3-second latency of the responses. MFRs acknowledged the naturalness of determining the physiological states of the VP through verbal communication, for example, with questions such as “Where does it hurt?” However, the question-answer dynamic in the verbal exchange with the VP and the lack of the VP’s ability to start the verbal exchange were noticed. Noteworthy insights highlighted the potential of domain-knowledge prompt engineering to steer the actions of MFRs for effective training.

**Conclusions:**

Generative AI in VPs facilitates MFRs’ training but continues to rely on instructions for effective verbal interactions. Therefore, the capabilities of the GPT-VP and a training protocol need to be communicated to trainees. Future interactions should implement triggers based on keyword recognition, the VP pointing to the hurting area, conversational turn-taking techniques, and add the ability for the VP to start a verbal exchange. Furthermore, a local AI server, chunk processing, and lowering the audio resolution of the VP’s voice could ameliorate the delay in response and allay privacy concerns. Prompting could be used in future studies to create a virtual MFR capable of assisting trainees.

## Introduction

### Background

**Figure 1:**
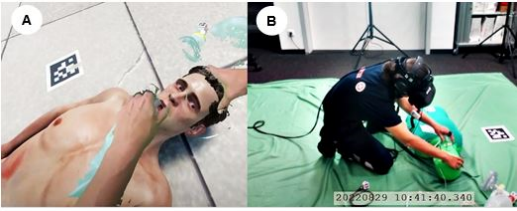
(A) The mixed reality view shows a 3D avatar that represents a victim of a traffic accident and a medical first responder’s real hands and tools, that is, an oxygen mask and an AMBU bag to provide treatment in an emergency scenario. (B) The green manikin and sheet on the floor in the real world enable chroma key compositing software to mix physical and virtual content in the mixed reality head-mounted display. A headset with headphones and a microphone provides a 3D sound environment and natural language communication capabilities with the virtual patient.

Virtual patients (VPs) are used in clinical scenarios and have features such as clinical information, case progression, and knowledge of diagnosis that are presented on digital displays; they are mainly used in health care services, education, or training to support the development of mental models in clinical reasoning [1]. VPs have lately been implemented in virtual reality (VR) and mixed reality (MR) simulations to study the training of medical first responders (MFRs) for the urgent assessment and treatment of victims, for example, in mass casualty incident (MCI) triage [2-4]. Particularly, a VP in MR was integrated in our previous study with chroma key compositing [2]. Chroma keying allows MFRs to interact with a physical manikin that is overlaid in the virtual world with a 3D avatar representing the victim of an accident, that is, the VP (Figure 1). The tangible VP can then show different injuries, movements, and facial expressions that match speech production, respiration patterns, or pain sounds. These sensory cues are crucial to determine the treatment or triage category that needs to be provided to the victim. For instance, if breathing difficulties are present or if there is an inability to follow simple commands, the patient is assigned a red category for immediate treatment [5,6]. Moreover, MR allows MFRs to see their own hands, bodies, and medical tools through the MR head-mounted display (HMD) as shown in Figure 1A, while providing haptic feedback with physical objects as shown in Figure 1B.

Training in medical emergency assessment and treatment for triage in MR simulations benefit end users and organizations alike because they are highly immersive and efficient when compared to existing real exercises, which sometimes involve hundreds of people, including actors, participants, and organizers [3]. Training in digital simulations increases its availability to more MFRs, simplifies the architecture and access to training facilities, and eliminates the need for human role players and props.

Notably, communication training is a major objective both in digital and in real-world medical emergency simulations. Developing communication skills is an important component of simulations that has shown to positively affect patient outcomes [7,8]. In real-world exercises, human role players act as injured persons or standardized patients, and MFRs interact with them in small groups in different scenarios. MFRs repeat this procedure several times to build communication skills. Ideally, retraining workshops should take place at least once a year to account for triage accuracy and overall performance drop [9].

Human social interaction is essential for verbal skill development. Nonetheless, real-world simulation and role-playing are costly and require intensive planning of infrastructure and mobilization and coordination of multiple personnel [10]. However, training verbal interactions in MR simulations for MFRs also comes with limitations and inherent problems. Social communication in digital simulations, and lately in MR, is trained with virtual agents, for example, avatars or conversational agents (CAs), that represent the victims in the simulations, that is, VPs. However, the verbal (written and spoken) interactions with VPs depend mostly on the state of the art of language technologies and thus have mostly been studied in health care with CAs that use scripted conversational flows or basic machine learning models [11].

In this context, the latest advancements with large language models (LLMs) and generative artificial intelligence (AI) allow low latency generation of sentences based on relatively small amounts of tailored contextual information to provide natural spoken interactions with computer systems for specific use cases, that is, prompting generative voice agents (GVAs) like ChatGPT [12]. However, ChatGPT can be computationally expensive and untrustworthy [13]. The question is whether we can use the performance and productivity of GVAs as an alternative for human role-players and previous VP technology in MR simulations to train MFRs. First, we must study the usability of GVAs in the context of VP training to find out how MFRs interact and perceive them. Hence, in this paper, we pose the following research questions (RQs): (1) How usable are verbal interactions with a GVA-based VP in MR emergency assessment and treatment training of MFRs? (RQ1) and (2) What is the overall perception of the GVA-VP’s voice quality simulating a victim of an accident? (RQ2)

Answering these questions required us to integrate ChatGPT’s verbal interaction capabilities in a previously studied MR medical emergency training VP. Following that, it was necessary to collect MFRs’ data about the treatment of patients during emergency treatment in accidents to design and test a prompt that derived accurate responses from the GVA-VP. Finally, we conducted a formative evaluation of the feature. Consequently, this work can guide effective prompt engineering, generative AI interaction design, and optimization of GVAs in MR training for MFRs.

### State-of-the-Art

#### Advantages and Disadvantages of CAs

CAs are used in a variety of health care applications, but conventionally, their implementation ranges from simple, specific domain knowledge and preprogrammed conversation flows to smart, predictive machine learning models that do not reach the spontaneous and somewhat improvised human-level communication abilities [11,14]. This inherent characteristic of traditional CAs is a limitation to the development of more realistic, natural verbal interactions. Therefore, CAs are mostly delivered through text-based mobile devices apps, web or desktop-based software for command-response, question-answer use cases in medical practice, for example, to support patients in handling information like scheduling appointments or giving test results, and, to a lesser degree, health care professionals with various degrees of accuracy on education and training; triage; diagnosis of respiratory issues; mental health problems, eating disorders, and sexual health problems [14,15]. However, even with restricted language communication ability, VPs have shown moderately effective results in medical education when screen-based, virtual learning environments (VLEs) or similar included instructional interventions and postactivity human feedback are used [16-18]. In training involving VLEs, VPs supported the improvement of verbal communication skills of students mostly in history taking and delivery of bad news, procedural skills, and clinical reasoning, and also of usability and satisfaction ratings [17]. The drawbacks in the usability of CA-based VPs and VLEs in real-world contexts include poor understanding because of limited vocabulary, voice recognition accuracy, error management of word inputs, general repetitive interactions, and lack of variability in conversations [18,19].

#### Advantages and Disadvantages of GVAs

The recent availability to the public of generative AI architecture integrated with LLMs, such as ChatGPT, provides potential ways to ameliorate the limitations of previous CA-based VPs with more natural verbal communication between humans and computer systems [12,20]. LLMs are trained on vast amounts of data that humans have generated in digital form throughout the years. These data allow LLMs to perform natural language processing (NLP) tasks, that is, humanlike text generation by predicting the likelihood of a word based on the previous one, context understanding, answering questions, language translation, or sentiment analysis [12,13,20]. GPT, a specific case of LLM, can further extend its functionality beyond NLP tasks. For example, Dall-E is based on GPT-3 and performs image generation tasks with text input, and ChatGPT-4 accepts images as input to generate text, leveraging computer vision, image recognition, and NLP [20,21].

Gaming studies and industry quickly understood the potential capabilities of GPT models in their fields. Nonplayer characters (NPCs) powered by GPT models are now capable of more engaging and natural verbal interactions, that is, written and spoken, with players. The history of the interactions is also stored in the memory of GPT models to create dynamic behavior of NPCs based on previous exchanges and contexts [22]. Furthermore, the personality of the characters can also be reinforced with modeled voices that match a desired role [23]. Hence, enabling NPCs with more natural conversational interactions can also be applied in MR triage training. To the best of our knowledge, there are currently no implementations or studies of GPT in MR training for medical emergency assessment and treatment, for example, triage training for MFRs. Furthermore, OpenAI ChatGPT has public access through an application programming interface (API) that is well documented and allows configuration of the prompt, language, voice quality, and other parameters [24].

However, a negative side of ChatGPT models is that they are computationally expensive, that is, they require billions of parameters to process prompts and generate responses. Consequently, responses take a short time to be produced depending on the constraints given in the prompt, the size of the answer, the traffic on the server, and other factors. Some of these elements can be optimized through OpenAI’s API, but the delay is always noticeable. Another drawback of ChatGPT is its proneness to fabricate facts, that is, hallucinations. This issue negatively affects its trustworthiness when factual information is needed. Finally, the use of ChatGPT represents a security and privacy risk because information is sent to remote servers for processing. These are 3 general limitations of ChatGPT, but several others exist [12,13,21].

There are, nevertheless, approaches to solve the delay produced by ChatGPT’s response processing time, its proneness to fabricate inaccurate responses given its probabilistic nature, and the security risks from remote data processing. For instance, breaking spoken sentences into chunks for transcription, that is, incremental automatic speech recognition (ASR), produces faster results in conversational AI [25]. More importantly, looking back into CAs’ preprogrammed conversation flows gives insights into synergizing the capabilities of CAs and GVAs to create voice agents. Incorporating knowledge graphs and ontologies, that is, structured data with labeled meaning for predefined decision trees found in CAs, to work in parallel with a GVA’s model, could accelerate the response time and enable the control of responses’ content when necessary [26]. Furthermore, since the release of Meta’s open-source LLM GPT, many new alternatives to ChatGPT have emerged. These fully functional GPT models perform similarly to ChatGPT, can run locally on home computers, and can be stripped down to any use case’s knowledge base [27].

## Methods

### VP MR Scenario

**Figure 2:**
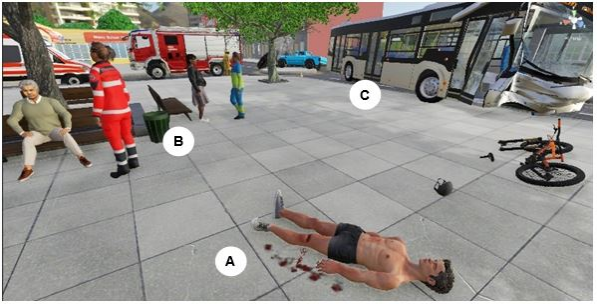
The accident scene presented to the medical first responders. (A) The overlaid 3D avatar simulating the victim of an accident, that is, the virtual patient. (B) Additional nonplayer characters that were already being attended to by virtual medical first responders. (C) Highlighted vehicles, buildings, and other objects for visual context.

The main audiovisual stimulus consisted of a VP representing a male victim of a traffic accident in a 3D scene made with Unity3D 2022.3.7f1 LTS. The scene consisted of a city’s street intersection where the aftermath of a traffic accident included crashed vehicles, MFRs, and victims (Figure 2). This scenario was the result of several workshops with MFRs from different emergency medical service (EMS) organizations across Europe as part of the project Med1stMR [2]. The 3D scene was presented to the MFRs through the Varjo XR-3 HMD, which used chroma key compositing to display virtual elements on a physical green manikin lying on a green sheet on the floor. MFRs could see an avatar overlaid on the manikin as well as real-world elements if they were not green, along with their own hands, body, and medical tools (Figure 1). Vive Trackers (version 3.0) were placed on the head, hands, feet, and groin of the manikin and mapped to the corresponding parts of the VP’s avatar. This allowed the MFRs to freely move the manikin and thus the VP. Both the green screen composition and tracking provided an immersive, tangible MR experience. To start the scenario, a bleeding cut on the VP’s left leg indicated priority for treatment. MFRs wrapped and secured a real tourniquet above the upper section of the wound after which the bleeding stopped. MFRs could communicate verbally with the VP at any time. Further visual inspection of the naked torso of the VP’s avatar revealed a hematoma on the right side of the chest. At the same time, the MFRs could lift the left hand of the VP to see the values on a pulse oximeter on the middle finger indicating a normal state of the VP’s heart rate at around 90 bpm and oxygen saturation at approximately 95%. When close to the VP, the MFRs could hear the VP’s groans and sounds of abnormal efforts to breathe. Sounds of pain were also automatically reproduced when the MFRs touched the wounded areas. At this point, MFRs would perform auscultation and hear rales with a real stethoscope. The VP’s skin coloration then started to turn blue, and the values of the pulse oximeter changed accordingly, that is, the heart rate started to rise and oxygen saturation dropped. At the same time, the VP started closing its eyes, no longer emitting sounds or responding to verbal interactions. MFRs then provided the VP with manual ventilation using a real resuscitator bag, which caused the VP’s oxygen saturation, heart rate, skin coloration, and verbal responsiveness to become re-established. At this moment, the MFRs were informed that the scenario had ended.

### GPT Integration

#### Overview

**Figure 3:**
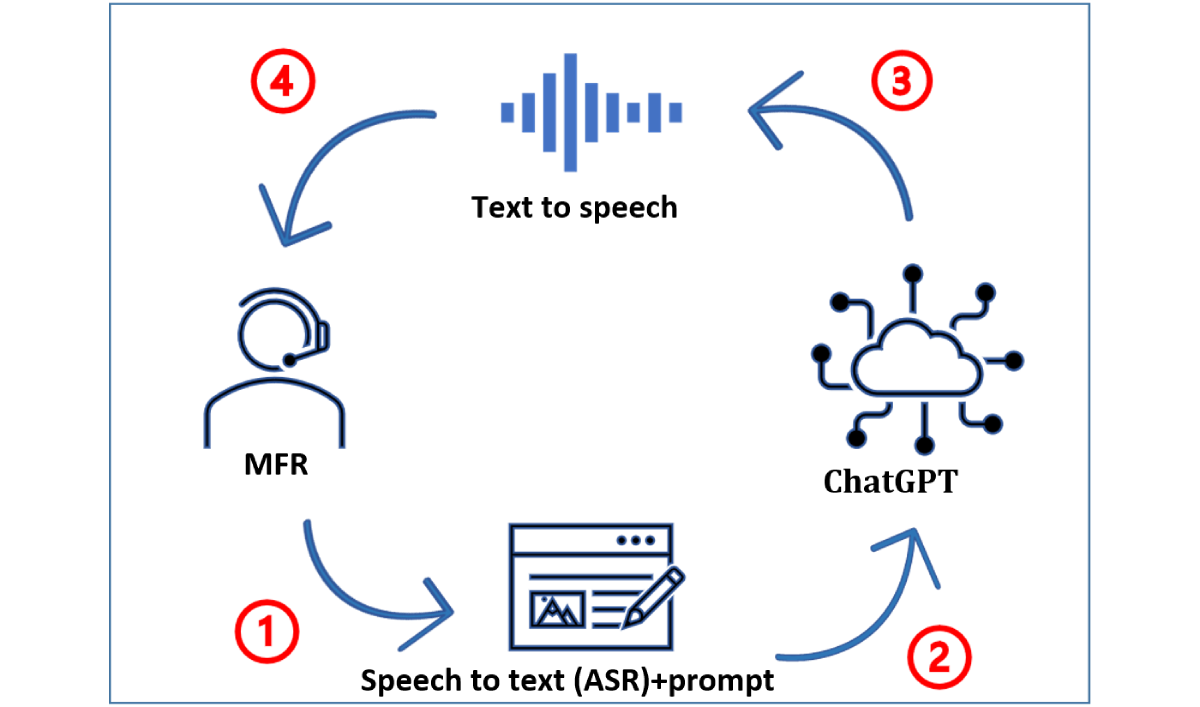
(1) The microphone picks up the voice of the medical first responder (MFR) and the automatic speech recognition (ASR) module transcribes it. (2) The text message is then added to the prompted attributes of an accident victim and both are sent to the ChatGPT model. (3) The generative voice model estimates the likelihood of the sequence of words to generate a written answer given its training data. (4) The answer is converted to audio by the text-to-speech engine with the characteristics of the modeled voice and then is output to the MFR.

Verbal interactions were enabled on the injured VP’s avatar with the OpenAI ChatGPT model (GPT-3.5 Turbo), which was used as the central AI response generator. The Unity virtual environment communicated with the cloud-based ChatGPT’s generative AI model with an adapted asset that wrapped ChatGPT’s APIs using C# scripts. This asset exposed ChatGPT’s settings in the Unity Editor using a private API key and allowed it to set ChatGPT’s model, voice, prompt, temperature, etc. Figure 3 shows a diagram summarizing the processing chain of a common ChatGPT implementation.

The steps summarized in Figure 3 are explained in detail to provide a better understanding of the technical and design aspects of the implementation and potential optimizations in the communication between the MFRs and the VP.

In the first step, the MFR’s voice is initially picked up by a Microsoft LifeChat LX-3000 headset with a noise-canceling, switchable microphone. The microphone is placed in front of the mouth and accurately triggers recording with the first utterances when a set threshold in Unity’s interface is reached. This headset is also lightweight and comfortable to wear. The recordings are sent for processing to OpenAI Whisper, a versatile multilanguage ASR module capable of converting speech to text (STT). Whisper is an accurate STT engine, but sometimes it can output transcriptions that are unrelated to the audio signal, that is, hallucinations. For example, it can pick up the sound of a hand hitting a desk and translate it into Korean characters or phonemes of the English language [28,29].

Next, the generated text message is added to the voice agent’s role characteristics that have been previously set in ChatGPT’s prompt. The prompt is a constraining feature of the model. The app developer allows to specify required responses. For example, the emotional state of the voice agent can be specified in the prompt, for example, “Play the role of a sad person.” Therefore, the model will generate text according to the provided emotion. Otherwise, ChatGPT can produce more open and undesired responses. Prompting is a reiterative process, and it can be tailored to a high degree. There are currently several guidelines for prompting ChatGPT with the goal of getting more accurate responses [12,21]. The ChatGPT Prompt section provides details on the prompt used in this study for the VP.

The 2 pieces of information, that is, the MFR’s message and the prompt, are used by the cloud-based GPT model to generate a text-based response grounded on the likelihood of the sequence of words. ChatGPT has been trained with large amounts of text (LLM) and will respond by matching the criteria given by the user’s message, the prompt, and the temperature setting [21]. The model used, GPT-3.5 Turbo, is trained with data up to 2021 [13,21].

In the final step, a different module, that is, ElevenLabs, generates audio streams from ChatGPT’s written response. ElevenLabs’ text to speech (TTS) engine is also responsible for the voice quality, for example, the pitch, emphasis, and timing of the artificial voice. This TTS module has several options that can be accessed from its API: male and female voices, stability, and clarity. Nonetheless, ElevenLabs also has several limitations. The voice models it provides were designed for public presentations, and therefore are energetic and upbeat. The voice of a person whispering or crying is currently not possible to recreate artificially with ElevenLabs [23]. Refer to the Voice Quality Model subsection for details on the construction of the VP’s voice.

ChatGPT was prompted with characteristics of a traffic accident victim collected from MFRs’ studies and generated corresponding responses during spoken interactions. The voice quality was modeled to match the role and accompanying abnormal breathing sounds and groans.

#### ChatGPT Prompt

The GPT’s prompt was designed based on previous studies with MFRs and their descriptions of role-playing, triage systems, and victim’s characteristics in MCIs [30]. These specifications about triage systems, physiological signals, behavior, and injuries were prompted in ChatGPT. Furthermore, to avoid GPT’s hallucinations, constraints were also prompted to only use the local language (German) and to respond “I don’t understand” to incomprehensible verbal commands or questions. The specific prompt was formulated in the German language, and we present the English translation as follows:

Your name is Tobi and you are a 28 years old male victim of an accident. A bus hit the corner of a building after avoiding two cars that crashed in the nearby roundabout. You were ridding your bicycle with your girlfriend and the bus hit you. You were going to the park to meet some friends. Your girlfriend was ridding next to you. You don’t know where your girlfriend is now. You have been lying on the ground for 20 minutes. You are now receiving assistance from medical first responders. You can hardly breathe, and it does not get better. You cannot be cured now. You do not feel good. You cannot feel good. You have to go to the hospital for immediate attention. You have a painful wound in your right ribs. You can hardly move. Your right leg is bleeding. Your vision is blurred. It is not possible for you to stand up or walk. You feel weak, tired, cold, sleepy, and drowsy. You have no allergies. You are not taking any medication. Respond only in German. You only understand German. Respond only by stuttering. Use only one 8-word sentence or less to respond. Stutter: “My ribs hurt” every minute. Never say: “Thank you.” Never answer: “Thank you.” If you don’t understand something, only answer with a stutter: “What?” or “I don’t understand you,” or “What are you saying?” Respond only by stuttering a maximum of an 8-word sentence. You are scared and anxious. Cry every minute: “Arrghh” or “Oh God help.” Respond only as if you were this character.

#### Voice Quality Model

The ElevenLabs AI voice was trained using our own recorded speech samples in English with prosody matching the simulated victim’s condition, that is, a heavy breathing and voice conveying pain. We input 25 uncompressed WAV files of around 55 second duration and high quality (48 KHz sample rate and 32 bits) into ElevenLabs web interface to train the voice synthesizer. A short part of the speech samples containing heavy breathing and groans was looped during the verbal interactions with the VP to fill the gap of ChatGPT’s response delay. However, even though the synthesized voice had a tame quality to it, it did not fully reflect a state of pain and suffering. This was expected given that most current TTS models target public speakers’ use cases for presentations, and thus, their models lack capabilities to whisper or cry. Stuttering instructions were added in the prompt to provide a suffering characteristic to the voice. The English-trained voice produced a foreign accent when the German-language prompt was applied to the ElevenLabs English version 1 model, which is normal in the context of international cities.

### Study Procedure

**Figure 4:**
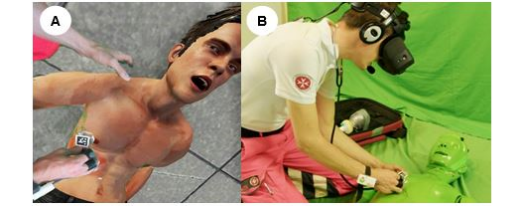
(A) The overlaid 3D avatar simulating the virtual patient (VP), the victim of an accident, with generative voice, breathing sound patterns, and groans. (B) A participating medical first responder talking to the VP using a noise-canceling microphone that helped to focus on the verbal interactions. Responses from the VP and other spatialized sound elements, for example, background conversation, the city environment, and the VP’s groans were reproduced through headphones.

Participants consisted of 24 MFRs from different EMS organizations from Austria and Germany. They were recruited from a network of European EMSs as part of the Med1stMR project [2]. Consent for audiovisual capturing and participation was obtained from the participants invited for the study. The MFRs were then equipped with the HMD, headphones, and microphone and went through a short visual calibration process. They took a minute to familiarize themselves with the environment while they were introduced to the scenario previously described. The VP’s delay of responses was also explained to the MFRs and then they were instructed to approach and interact with the VP just as in real life using nonscripted, spontaneous natural language communication (Figure 4). The verbal interactions consisted of the MFRs’ own questions and instructions associated with emergency training. MFRs treated the VP according to the wounds and audiovisual feedback they received. When the MFRs felt ready, they stopped interacting. The MR experience was performed once and lasted from 6 to 10 minutes.

Afterward, version 2 of the Mean Opinion Scale-Expanded (MOS-X2) questionnaire was used to study the participant’s perception of voice quality. The Subjective Assessment of Speech System Interfaces (SASSI) questionnaire was used to question the MFRs regarding the usability of the voice interactions. Open-ended questions and conversations with the participants were conducted after completing the questionnaires. Notes were made during this process where participants reflected on their experience with the GVA. Each participant took approximately 30 minutes to complete the entire procedure. Audiovisual screen captures were recorded as well. The design of the experiment for the assessment of a GVA’s voice quality and the usability of the GVAs’ interactions was based on the existing evidence of studies using the same and similar methods, for example, the interactive short conversation test and the International Telecommunication Union quality of experience [31-33].

### Measurements

Two questionnaires were used to assess the user experience of the trainees: the MOS-X2 [34] for investigating the experience of voice characteristics, and the SASSI [35] for general voice-related user experience.

The MOS-X2 consists of 4 Likert items, that is, intelligibility, naturalness, prosody, and social impression. Each item had a scale from 1 to 10 (1=extremely unnatural and 10=perfectly natural).

The SASSI was used to assess the usability of voice interactions. SASSI measures 6 dimensions of usability, that is, system response accuracy, likeability, cognitive demand, annoyance, habitability, and speed, with 39 Likert items containing scales from 1 to 7, that is, strongly disagree to strongly agree.

### Ethical Considerations

All the studies within the MED1stMR project followed the ethical guidelines and procedures established at the beginning of the project [36]. The studies were approved by the ethics committee of the Faculty of Behavioural and Empirical Cultural Sciences at the University of Heidelberg, Germany (AZ BEU 2023), and managed by the appointed ethical advisor. Informed consent was obtained from all participants involved in all field trial studies conducted within the MED1stMR project, including the study described in this paper. Participants for this evaluation were local MFRs who were invited to participate as trainees via email. Participation of MFRs in this study was voluntary, and the invitation was sent to recruits through the different EMS organization networks and submitted for an ethics approval. They were informed that (1) the study would be recorded, transcribed, and analyzed; (2) their identity would remain confidential; and (3) that participation was voluntary and could be withdrawn at any time.

In addition, all researchers who were involved in the study were listed. This included the researchers’ names, their roles in the study or project, affiliations, and contact information (eg, email address and phone number). This step was necessary to establish the transparency of the research process as well as reflect who had access to the generated data. This information was also to be provided to the participants so that they could make a fully informed decision on whether they were willing to share their data with the parties involved. All partners in the Med1stMR project provided detailed descriptions of their study before it could be voted on regarding potential ethical concerns. Furthermore, an explanation of how the data are stored (eg, on a hard drive or secured server) and who will be able to access the data (and how) was specified in the consent form signed by recruits. Finally, the collection of potentially identifiable information (eg, video recordings or email addresses) was detailed. Video and audio recordings were scheduled for deletion after 90 days, and partner employees were also obligated to delete locally saved copies of these materials. The Austrian Institute of Technology is hosting a protected SharePoint server on its IT premises, located in Giefinggasse 4, A-1210, Vienna, for project data storage. The data are only saved on the local servers and not transferred to the Microsoft cloud.

## Results

We performed the study with 24 MFRs (women: n=4, 17% and men: n=20, 83%) aged 19 to 50 (mean 30.61, SD 9.21) years. Central tendency analysis was performed for 2 questionnaires.

### Perception of Voice Quality

**Figure 5:**
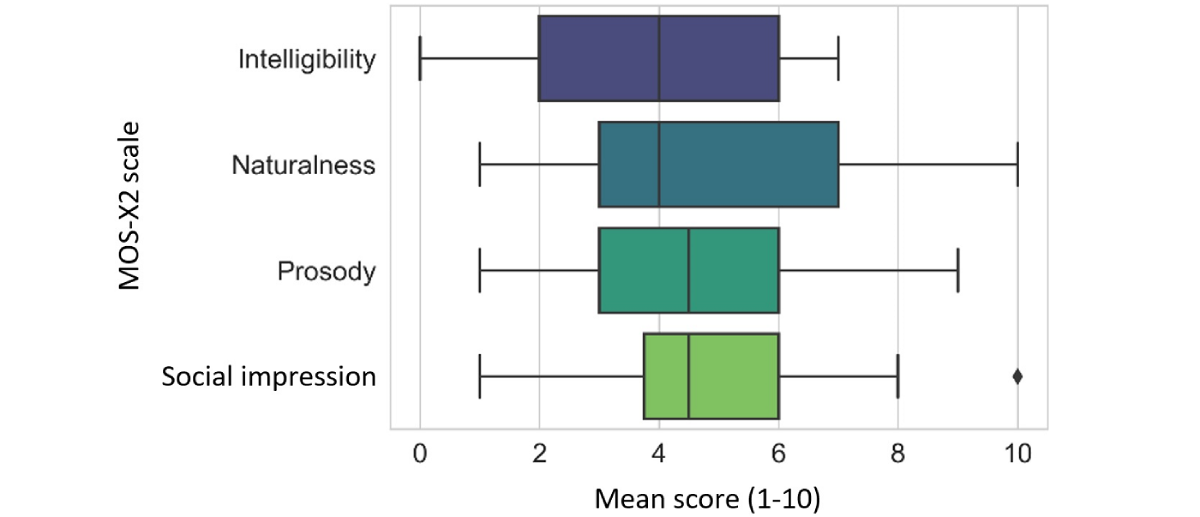
The Mean Opinion Scale-Expanded, version 2 (MOS-X2) rating scale measures the perception of voice quality. Overall, the 4 dimensions, that is, intelligibility, naturalness, prosody, and social impression, showed moderate ratings and high dispersions. The perception of the naturalness of the voice was rated the highest.

Results of the perception of the voice quality with the MOS-X2 questionnaire indicated a moderate humanlike perception of the GVA’s voice with high dispersion values. Intelligibility was rated with a median value of 4 (IQR 3.75-6), mode 4, and a dispersion with a range of 10. Naturalness was rated with a median value of 4 (IQR 3-7), mode 3, and range R of 9. Prosody was rated with a median value of 4.5 (IQR 3-6), mode 4, and range R of 8. Social impression was rated with a median value of 4.5 (IQR 3.75-6), mode 6, and range R of 9. A graphical representation of these results is shown in Figure 5.

### Usability of Voice Interactions

**Figure 6:**
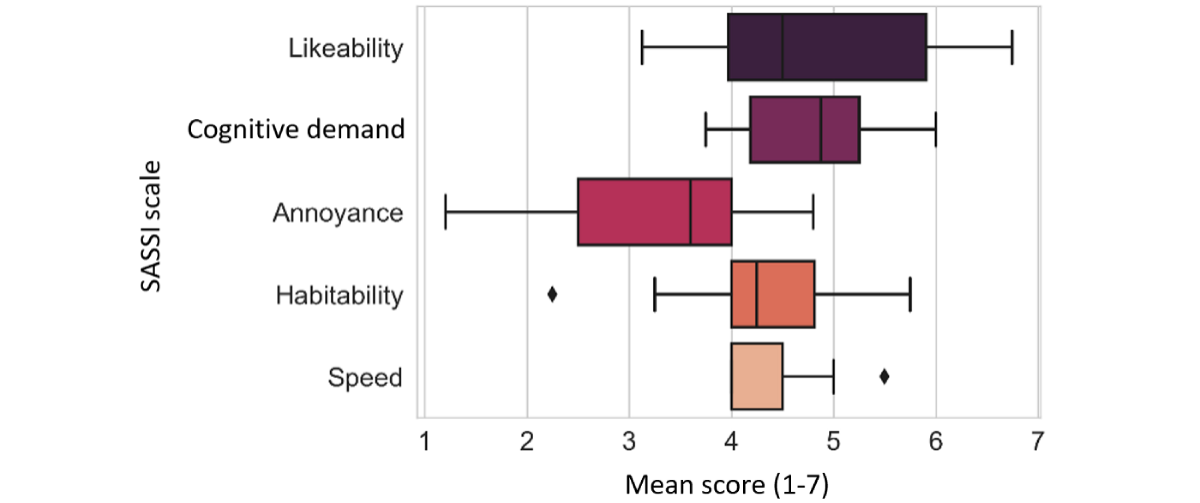
The Subjective Assessment of Speech System Interfaces (SASSI) scale measures the usability of the generative voice agent’s (GVA) interactions. All 5 dimensions show a moderately high usability of the GVA, especially on the likeability scale.

Results of the usability of the voice interactions with the (SASSI) questionnaire indicated Likeability was rated with a median value of 4.50 (IQR 3.97-5.91) and a range of 3.63. Cognitive demand was rated with a median value of 4.80 (IQR 4.20-5.40) and a range of 2.20. Annoyance was rated with a median value of 3.60 (IQR 2.50-4) and a range of 3.60. Habitability was rated with a median value of 4.25 (IQR 4-4.81) and a range of 3.50. Speed was rated with a median value of 4 (IQR 4-4.50) and a range of 1.50. A graphical representation of these results is shown in Figure 6. The usability measures indicated good overall interaction performance, but there is room for improvement in all 6 dimensions.

Both questionnaires supported the integration of ChatGPT in VPs for MR emergency assessment and treatment training and will be further discussed in the subsequent section. Similarly, the interviews with the MFRs and the analysis of audiovisual captures yield intriguing insights that merit further discussion.

## Discussion

### Principal Findings

#### Overview

The results show mixed experiences of the MFRs with the GVA-VP. On one hand, many participants were impressed and excited about the idea of interacting with a virtual agent, not only because of the novelty but also because it enabled training without the need for role players. On the other hand, several technical limitations still stood in the way of a completely seamless social experience. Accordingly, we divided the Discussion section into 2 parts—verbal interaction and technical insights.

#### Verbal Interactions Analysis

MFRs varied greatly in their communication approaches. In total, 5 (21%) of the 24 MFRs had very few verbal interactions with the VP, but 3 (12%) MFRs detailed every step of their assessment and treatment and were continuously communicating with the VP. Furthermore, 3 (12%) MFRs approached the VP with the question “Can you hear me?” to which the VP answered with variations of “AAhh. Pain. Hospital. Help. Please. Treatment.” or “Oh God help, I cannot breathe.” while exhibiting great breathing efforts. Even though the participating MFRs were explained about the delay of approximately 3 seconds for the GVA’s response, the verbal interactions usually had overlaps. The participants sometimes took a brief pause after their initial verbal interaction and then continued with the second question expecting the GVA to answer quickly. Given that ChatGPT takes 2 to 3 seconds to respond, the participants’ second question thereby overlapped with ChatGPT’s response. During these overlaps or when the microphone picked up noises, for example, tools hitting the microphone, ChatGPT produced sentences like “I don’t understand, please help.” as instructed in the prompt to manage errors of word inputs. This created some confusion among participants while taking turns to speak and was reflected in the results of the SASSI questionnaire. For instance, the participants’ rating was close to 5 (out of 7) in habitability (the match between the language that humans use and the language that the computer accepts) and speed (the VP’s response time), indicating that sometimes the system did not do what the MFRs intended, they were not sure what the system was doing, or they noticed the slow response speed. ChatGPT’s response delay is the most crucial issue to solve, and a potential solution is discussed subsequently in this section.

In a few cases, the MFRs waited for enough time to get an answer and acted according to it. For example, 4 (17%) of the 24 participants asked the GVA either “What happened?” “Does it hurt? Where does it hurt?” or “Can you breathe well?” and waited for an answer. The GVA accurately responded “Accident with the bus,” “Yes, hurts, right ribs,” and “I cannot breathe” correspondingly. Then, the MFRs continued inspection in the indicated or relevant area. This example shows the need for instructions and continuous monitoring to better manage turn-taking in the verbal interactions between the MFRs and the VP to fully exploit GPT’s current capabilities.

A word on the perception of the voice produced by the TTS engine is also necessary. ElevenLabs is a powerful and realistic TTS and voice cloning technology, but currently, it only provides voices for presentations like podcasts and advertisements. This will change soon, but we found that training ElevenLabs with a voice conveying suffering, breathing difficulties, and groans worked adequately to resemble a human victim with an injury on the ribs who has difficulties producing speech. This was also the motivation to add stutters. These qualities are reflected in the MOS-X2 ratings regarding voice characteristics, which were rated moderately by the participants (eg, intelligibility and naturalness, both with a median of 4), which show room for improvement. A few MFRs mentioned the American accent of the German-speaking GVA as a factor that affected understanding, but 2 (8%) MFRs stated their positive perception of the accent. This may be a desirable feature since victims can have problems with verbal communication in real-world scenarios and this can be the reason for the higher scores of naturalness and likeability. The multicultural characteristic of intranational cities was a deciding criterion when choosing a voice with an accent. By contrast, the ElevenLabs Multilingual version 2 model produces natural German speech without an accent and can reflect the characteristics of the training voice samples with more accuracy, that is, it has a more natural voice when conveying pain. Sounds of sirens and NPCs talking in the background also have to be considered in a more in-depth communication training study of the subjective perception of the acoustic environment or soundscape and its potential influence on cognitive performance [37].

#### Technical Insights

Furthermore, as described in the Methods section, there are 4 processes involved in the use of ChatGPT for the generation of humanlike voice communication, that is, ASR or STT transcription, message and prompt analysis, textual response generation, and TTS transformation. Each of these processes has inherent difficulties that deserve a deeper analysis, but benchmarking our implementation of the GVA-VP will be part of a separate study. However, during this study, we identified ways of ameliorating the GVA-VP’s implementation process.

The first challenge relates to the characteristics of the or microphone and how this affects the OpenAI’s STT module, that is, Whisper, and thus the overall usability. Noise-canceling headphones or a silent environment guarantee that the GVA’s voice and the sound environment of the MR experience are adequately mixed for an optimal and realistic experience for the user. Moreover, the passive noise-canceling feature of headphones’ earcups prevents sound leakage from the speakers to the microphone. Similarly, a noise-canceling microphone helps to only pick up the voice required to be sent to the STT engine. Here, the threshold value at which the system starts recording speech is very important, because it must be leveled in accordance with the user’s breathing sounds that are also being picked up by the microphone and could confuse Whisper and trigger a response. Beamforming microphones in the body of the headphones could be a good solution. An easy-to-access button to activate or deactivate the microphone is also possible but may interfere with the MFR’s freedom of movement to perform treatment using their tools.

In the context of extended reality, encompassing mixed, virtual, and augmented reality, the spatialization of sound is a key feature. For instance, in MR triage training, users must get closer to the GVA representing the victim to hear its voice. This mimics real-world phenomena, such as distance attenuation and sound source localization. Such spatialized soundscapes encourage head movements, which can be spontaneous and abrupt. Consequently, it is crucial for the headset to offer a secure yet comfortable fit.

Furthermore, ChatGPT prompting displayed some common challenges also present in this work: the prompt must always be very specific to avoid inaccurate responses that are generated because of unintended noises or imprecise requests. Humans, in particular MFRs, can behave in unexpected ways and the GVAs must be provided with a fallback mechanism to respond. This can be as simple as responding “I don’t understand” to incomprehensible verbal commands or questions as previously described, but it is possible to react differently to different types of sounds, for example, responding “What?” when mistakenly misunderstanding a given language accent or pronunciation, and responding “Say again” when answering to out-of-context questions or commands. This can add further variability and realism to the interactions.

Finally, a discussion about the implementation of knowledge graphs and ontologies is important because it has the potential to serve as a robust parallel voice generation mechanism that provides quick, accurate, genuine, and verifiable information. When we abstract from the implementation of GVAs in MR medical emergency training, we can see that the fundamental problem with AI is that we do not know if what it is saying is accurate, because there are no tools developed to understand why it reaches a particular decision, that is, the well-known problem of AI explainability. The common understanding is that AI functionality is years ahead of explainable AI. ChatGPT can produce responses that contain false information. While this was not a concern in our study, it is a major problem for future GVAs and should be attended to. The integration of ontologies in GVAs then adopts a crucial role; however, there is more. The domain of MCI has no general formalization or standards. There are many triage systems to prioritize the victims’ need for resources, but organizational and staff interoperability is lacking.

### Limitations and Future Studies

While an important and original contribution of this paper is showing the MFRs’ experience, the usability of GPT-based AI in a VP, and its capacity to resemble human communication for MR medical emergency training, the goal to achieve a humanlike verbal interaction still needs a lot of work. GPT verbal interactions are still limited by a noticeable latency, reduced availability of voice prosody, natural conversational turn-taking, and autonomous speech generation strategies. This study was very helpful in identifying these drawbacks of the implementation with MFRs, who have immensely contributed to the road map of realistic GVAs for MCI simulations. Future studies will include testing an improved version of the GVA-VP inside the MR experience.

Therefore, we plan to implement a local AI server with optimized domain-knowledge LLM for faster response times. It is then necessary to further collect MFRs’ requirements to construct an ontology for an outlook of how an MCI’s knowledge base formalization could work. Once an ontology is in the standardized Web Ontology Language format, it can be visualized with tools like Web-Based Visualization of Ontologies and connected to other systems for scalability to extend the potential of MFR training. Ontologies can support the functionality of GVAs by reducing the response delay and providing accurate answers. For example, a virtual MFR using GPT could be integrated with domain-knowledge standards to assist human MFR trainees.

### Comparison With Prior Work

To the best of our knowledge, there are no studies of GPT implementation in MR simulations for medical emergency training of MFRs. Baetzner et al [3] presented a review of immersive VR (n=3) and MR (n=1) studies and their effectiveness in preparing MFRs for crises, but there is no mention of verbal communication or verbal interactions with VPs. Real Response Blueroom [4] is a commercial application of physical manikins integrated with sensors and humanlike features, for example, bleeding, to resemble a victim without a voice within a VR environment. In our previous study, we used a similar setup with a green torso overlaid with a 3D avatar to study tangible interactions in MR and used human role players as voice actors to communicate with the MFR trainees [2]. Moreover, there is a wide variety of VP studies dating back over a decade that ranges from pen and paper, screen-based questionnaires and avatars, and more recently, rule-based and basic machine learning CAs in text or voice apps on mobile devices or in smart speakers [1,14-19]. Overall, the VR and MR studies show their cost-efficient, reproducibility, safety, and immersion benefits, and recent CAs in VP studies have shown moderately effective results in medical education and services of different types but not in medical emergency training for MFRs [14-18]. Similarly, as shown in the Introduction section, state-of-the-art CAs show poor understanding because of limited vocabulary, voice recognition accuracy, error management of word inputs, general repetitive interactions, and lack of variability in conversations [18,19]. This paper supports some of those previous findings and shows the potential to overcome conventional CAs’ drawbacks with LLM’s rich vocabulary, high voice recognition accuracy of Whisper, good capabilities of error management of word input, and variability in responses with prompt engineering.

### Conclusions

A GVA based on OpenAI ChatGPT was integrated into the 3D avatar of a VP that represented a victim in an MR simulation for medical emergency assessment and treatment training of MFRs. The perception of the GVA’s voice quality and its usability were studied with MFRs to determine if the artificial voice agent could be effectively used in emergency training and if its voice quality matched that of an accident victim. The results showed that the MFR participants had a moderately high perception of naturalness for the GVA’s voice quality and equal likeability perception for the GVA’s usability. Moreover, voice quality measures of intelligibility, prosody, and social impression appropriateness were moderate, pointing to paths of improvement and deeper analysis, for example, how to extend the STT system’s ability to cry, whisper, or moan or determining if a victim with high intelligibility is desirable in emergency training. Therefore, it is reasonable to conclude that the GVA was usable in MR medical emergency training and resembled a victim of an accident to a moderate degree. Furthermore, the usability of the GVA was accurate due to its state-of-the-art capabilities; it was engaging and required an appropriate perceived level of cognitive load. Reports of delays in responses and overlapping verbal interactions indicated a need for a faster system and the development of conversational turn-taking strategies. We can then further support previous findings that showed the need for instructional interventions to fully take advantage of the usability of GVAs in medical emergency training of MFRs. This study constitutes a novel contribution to MR and MCI triage training, describing a system that potentially performs better than state-of-the-art CAs, which have limited communication capacity and thus are deterministic and unnatural.

The use of GVAs to replace role-players in MR training offers numerous advantages. Costs are significantly reduced as there is no longer a need to hire and train human role-players, and at the same time, this increases scalability to simulate a larger number of patients with injuries and thereby helps accommodate multiple participants. Virtual agents ensure consistent performance and enable standardized training experiences. They can be customized to simulate different characters and behaviors, and scenarios can be tailored to specific training objectives. In addition, GVAs provide a safe environment for trainees, enable objective performance evaluation, and support repetition and iteration. These benefits improve the effectiveness, efficiency, and accessibility of MR training programs.

## Abbreviations

AI: artificial intelligence
API: application programming interface
ASR: automatic speech recognition
CA: conversational agent
EMS: emergency medical service
GVA: generative voice agent
HMD: head-mounted display
LLM: large language model
MCI: mass casualty incident
MFR: medical first responder
MOS-X2: Mean Opinion Scale-Expanded, version 2
MR: mixed reality
NLP: natural language processing
NPC: nonplayer character
RQ: research question
SASSI: Subjective Assessment of Speech System Interfaces
STT: speech to text
TTS: text to speech
VLE: virtual learning environment
VP: virtual patient
VR: virtual reality

## Data Availability

The datasets generated or analyzed during this study are available from the corresponding author upon reasonable request.
